# Hypocrellin: A Natural Photosensitizer and Nano‐Formulation for Enhanced Molecular Targeting of PDT of Melanoma

**DOI:** 10.1002/wnan.1997

**Published:** 2024-11-20

**Authors:** Precious Winterrose Gugu Nkosi, Rahul Chandran, Heidi Abrahamse

**Affiliations:** ^1^ Laser Research Centre, Faculty of Health Sciences University of Johannesburg Doornfontein South Africa

**Keywords:** hypocrellin, melanoma, nano‐formulation, photodynamic therapy

## Abstract

Nano‐formulation has generated attention in the battle against cancer, because of its great flexibility, reduced adverse side effects, and accuracy in delivering drugs to target tissues dependent on the size and surface characteristics of the disease. The field of photodynamic treatment has advanced significantly in the past years. Photodynamic techniques that use nano‐formulations have surfaced to further the field of nanotechnology in medicine, especially in cancer treatment. The pharmaceutical industry is seeing a growing trend toward enhanced drug formulation using nano‐formulations such as liposomes, polymeric nanoparticles, dendrimers, nano‐emulsions, and micelles. Natural extracts have also shown adverse effects when employed as photosensitizers in cancer therapy because they are cytotoxic when activated by light. Still, natural photosensitizers are a big part of cancer treatment. However, some shortcomings can be minimized by combining nano‐formulations with these natural photosensitizers. The synergistic improvement in medication delivery that maintains or increases the mechanism of cell death in malignant cells has also been demonstrated by the combination of photodynamic therapy with nano‐formulations and natural photosensitizers. Lastly, this review assesses the feasibility and potential of a photodynamic therapy system based on nano‐formulations and natural photosensitizers in clinical treatment applications and briefly discusses the removal of toxic compounds associated with nano‐formulations within cells.

## Graphical abstract

Nanotechnology and combination with natural photosensitizers are a prospective means of controlling cancer. Studies on photodynamic therapy (PDT) have shown that improvements in drug administration are required in order to preserve or enhance the mechanistic cell killing effects of PDT on malignant cells. As a promising method of treating cancer, nano‐phototherapy has shown mechanism of cells death, including necrosis, autophagy, and apoptosis.

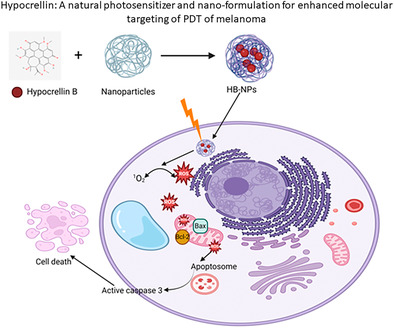

## 1 Introduction

Melanoma is a type of skin cancer that arises from the transformation of melanocytes, or the cells that produce the color melanin (Swavey and Tr 2013) Melanocytes are basal layer epidermis cells generated from the neural crest that are found in uvea, skin, hair, mucosal epithelia, and meninges. Melanocytes' main job is to make melanin inside melanosomes and communicate meaning to nearby keratinocytes through dendritic processes. Eumelanin and pheomelanin, the two types of melanin pigment produced by melanocytes, are both from precursor tyrosinase. The development of melanoma can be aided by several factors, including exposure to ultraviolet radiation (Rastrelli et al. 2014). Depending on where they live, people of the same ethnicity have varying incidences of melanoma. The amount of incident UV radiation varies depending on factors such as seasonality, cloud cover, latitude, altitude, and atmospheric absorption. Melanoma pathogenesis may be influenced by genetic factors. Uhara et al. discovered the BRAF mutation in melanoma patients who did not have long‐term UV exposure. Numerous investigations have revealed that BRAF, a serine/threonine protein kinase linked to RAS–RAF–MEK, has mutations between 40% and 50% of cutaneous melanomas (Poynter et al. 2006). An extracellular signal‐regulated kinase (ERK), one of the most prevalent mutant isoforms of cancer, is phosphorylated with BRAF activation (Poynter et al. 2006). Melanoma can spread into other parts of the body leading to metastatic melanoma and the outlook for this type of disease might be dismal. The predicted 5‐year survival rate is 10%, with much worse prognoses for distant metastases than for local dissemination. The definitive treatment for primary melanoma involves either wide excision surgery or Mohs micrographic surgery, which involves surgical removal of the tumor. Late‐stage melanoma has high genetic diversity, can be challenging to cure metastasizes, and is resistant to treatment (Eisen et al. 2006). However, innovative targeted immunotherapies have considerably extended survival times and improved quality of life for patients with metastatic melanoma. Photodynamic therapy (PDT) is one of the promising alternative forms of treatment for melanoma, and it is crucial to conduct studies aimed at advancing it. Other therapies such as the combination of phototherapy and immunotherapy have also shown promising results on metastatic melanoma.

Melanoma incidence has progressively increased globally (Figure S1) during the last few decades (Erdmann et al. 2013; Globocan 2013; Linos et al. 2009; Whiteman, Green, and Olsen 2016). Annual incidence has risen by up to 4%–6% in several fair‐skinned populations, particularly in North America, Northern Europe, Australia, and New Zealand (American Cancer Society [ACS] 2017a, 2017b; Nikolaou and Stratigos 2014). Incidence rate increase varies greatly across ethnic groups and geographical regions, as well as across age and gender (Apalla et al. 2017; Erdei and Torres 2010; Globocan 2013; Surveillance 2013; Whiteman, Green, and Olsen 2016). Australia and New Zealand have the highest recorded incidence rates globally, with up to 60 cases per 100,000 persons each year (MacLennan et al. 1992). From 2003 to 2007, white individuals in the United States only had rates of 25.4% for males and 16.9% for females (Kohler et al. 2011; Jemal et al. 2011). Despite accounting for less than 5% of all cutaneous malignancies, melanoma is responsible for the majority of skin cancer deaths (Ribas et al. 2015). In the 1990s, mortality rates in the United States decreased by 39% for women and 29% for males aged 20–44. However, rates soared by 70% and 157% for men aged 45–46 and greater or equal to years (Ries et al. 2004). Melanoma in the elderly may exhibit a unique biological behavior or changed host defense mechanism, leading to greater incidence and fatality rates. In addition to the significant burden on public health, the annual expenses of melanoma management are high (15). In the United States alone, the annual cost of melanoma treatment has increased by 288% in less than a decade. Skin cancer has an economic impact that includes both direct and indirect expenditures. Direct costs include the management of skin cancer from diagnosis to follow‐up, as well as the use of healthcare resources such as hospitals, medical, and allied health services. Indirect costs indicate the lost production caused by an individual's inability to work (morbidity costs such as sick leave and early retirement) and premature mortality (death before the age of 65, Australia's working age limit). As new, expensive pharmacologic treatments enter the market, expenses are likely to grow even faster. Melanoma accounts for $3.3 billion of the $8.1 billion in total direct skin cancer annual costs (Akushevich et al. 2013). Melanoma's indirect expenses are anticipated to exceed $3.5 billion per year (Ferlay et al. 2013). As the incidence and mortality rates rise, so will the expenses of treatment and indirect care (Erdmann et al. 2013). However, when more preventative techniques are introduced to address rising incidence, melanoma‐related expenses may improve, potentially reducing the economic burden by $2.1 billion per year (Garbe and Leiter 2009).

Decades of research have helped improve the understanding of melanoma's epidemiology, risk factors, and natural history, as well as a framework for prevention and control (Koh et al. 1995). Melanoma's public health burden could be decreased by effective primary and secondary prevention strategies. Excessive sun exposure (primary prevention) may theoretically lower the incidence because two‐thirds of melanoma cases are associated with excessive sunlight exposure (Armstrong and Kricker 1993). Individual behavior modifications (such as reducing ultraviolet exposure and utilizing protective clothing and sunscreen) can be used as primary preventative techniques, as can population‐wide policy and environmental interventions. Early detection (secondary prevention) should boost melanoma cure rates, as long‐term survival rates of over 95% for stage 1A melanoma drop dramatically to less than 5% in metastatic disease (Balch et al. 2020). Furthermore, these external, visible malignancies have recognized risk factors (which should be obvious to both the general public and health professionals) and can be effectively treated in their early stages (Rhodes 1995).

### 1.1 Melanoma Pathophysiology

As discussed above, melanoma develops as a result of the overgrowth of melanocytes and that is primarily caused by UV radiation and mutations within the DNA. The mutations contribute to different features of melanocytic neoplasia; however, some variants are designated driver mutations because they are more likely to trigger melanocytic transformation, the initial stage of tumor formation, progression, and dissemination. Vogelstein et al. and Shain et al. have effectively documented the genetic evolution that occurs during the progression of melanocytic lesion to malignant melanoma (Shain et al. 2015; Shain and Bastian 2016; Vogelstein and Kinzler 2015). First, a normal melanocyte receives initiating driver mutation, which causes melanocyte hyperplasia and nevi formation (Pollock et al. 2003; Poynter et al. 2006; Sensi et al. 2006; Shain et al. 2015; Shain and Bastian 2016). These phases are referred to as the breakthrough phase, and they involve low mutational burden and copy number changes (Sensi et al. 2006; Vogelstein and Kinzler 2015). Common alterations observed in melanocyte nevi are BRAF mutations (Poynter et al. 2006; Pollock et al. 2003; Sensi et al. 2006). Mutations in BRAF and NRAS are typically mutually exclusive, with NRAS mutation occasionally seen in nevi, particularly congenital nevi (Bauer et al. 2007; Martins da Silva et al. 2019). The following step is known as the expansion phase, in which some melanocytic nevi proceed to intermediate lesions and eventually develop into melanoma in situ, accompanied by the acquisition of TERT promoter mutations and high mutation burden (Chiba et al. 2017; Shain et al. 2015; Shain and Bastian 2016; Vogelstein and Kinzler 2015). The gene encodes telomerase reverse transcriptase, the catalytic component of telomerase, an enzyme necessary for telomerase maintenance (Chiba et al. 2017). Aberrant telomerase expression leads melanoma cells to replicative immortal (Chiba et al. 2017). Primary melanoma progresses to malignant melanoma after accumulating mutations in CDKN2A, TP53, PTEN, and genes encoding SWI/SWF chromatin remodeling complex compounds (Shain et al. 2015; Shain and Bastian 2016; Vogelstein and Kinzler 2015). This phase is distinguished by a high tumor mutational burden and increasing copy number changes (Shain et al. 2015; Shain and Bastian 2016). To note, about 20%–40% of melanomas start from nevi, while the rest are de novo; nonetheless, de novo melanomas may arise from clinically undetectable precursor lesions, which may follow a similar trajectory as detectable lesions.

In addition to the genetic abnormalities linked to metastatic melanoma formation, several important signaling pathways are dysregulated during melanoma advancement, including the WNT, MAPK, and PI3K/AKT pathways (Monteiro, Toricelli, and Jasiulionis 2015; Lopez‐Bergami, Fitchman, and Ronai 2008). These pathways have a role in melanoma cell proliferation, growth, survival, cell death evasion, and metastatic progression (Monteiro, Toricelli, and Jasiulionis 2015, Lopez‐Bergami, Fitchman, and Ronai 2008). Signal transduction through the WNT, MAPK, and PI3K/AKT pathways in melanoma cells affects the expression of cell adhesion molecules and peptidases, allowing for extracellular matrix (ECM) remodeling and cancer cell migration (Monteiro, Toricelli, and Jasiulionis 2015; Huntington et al. 2004; Lee et al. 2014; Yao et al. 2019; Hess et al. 2003). Melanoma progression is associated with increased matrix metallopeptidase (MMP) production and function. The MMP stimulates the breakdown of the ECM, which encourages melanoma growth during early stages and subsequent migration to distant organs (Monteiro, Toricelli, and Jasiulionis 2015; Hofmann et al. 1999; Hofmann et al. 2000). In addition to MMP‐cleaving links between melanoma cells and the ECM, loss of adhesion molecules such as integrins and cadherins contributes to melanoma cell motility from the original location (Damsky, Rosenbaum, and Bosenberg 2010; Hsu et al. 1996; Monteiro, Toricelli, and Jasiulionis 2015; Paluncic et al. 2016; Tucci et al. 2007). Cell adhesion molecules are necessary for cell attachment to the basement membrane as well as cell–cell interactions, which allow tissues and organs to form normally. Cell adhesion molecules, including integrins and E‐cadherins, are involved in the attachment of melanocytes to the basement membrane as well as the contacts between keratinocytes and melanocytes (Kim, Finlay, and Baguley 2013; Tang et al. 1994). During melanoma advancement, E‐cadherins are gradually reduced to allow for dissociation between melanocytes and keratinocytes, followed by simultaneous overexpression of N‐cadherins to enable melanoma cell survival and migration through tissues, a process mediated by the PI3K/AKT pathway (Hao et al. 2012; Lade‐Keller et al. 2013; McGary, Lev, and Bar‐Eli 2002). Alongside cadherin expression changes during metastasis, integrins can be altered to enhance motility and migration into hospitable metastatic environments by changing basement membrane contacts, promoting angiogenesis formation, and MMP production (Das et al. 2017; Jiao et al. 2012; Kumar et al. 2001; Monteiro, Toricelli, and Jasiulionis 2015) (Figure S2).

### 1.2 Types of Melanomas

Melanocytes grow abnormally in malignant melanoma, a skin cancer that can spread to other parts of the body if normal controls are not in place. Melanoma is therefore categorized into four different types by its histologic characteristics and incidence frequency (Figure S3).

#### 1.2.1 Superficial Spreading Melanoma

Superficial spreading melanoma is the most popular term used to describe malignant melanoma, which can affect individuals of any age. Though it can appear everywhere on the body, the legs and trunks of both men and women are usually affected. Usually, it has a greater diameter than 0.5 cm. It usually starts as an asymmetric epidemic that ranges in color from black to red, brown, blue, and white due to anomalies in the pigment pattern (Alasadi and Alsafy 2017).

#### 1.2.2 Nodular Malignant

It is one of the less common types but more malignant; it typically appears as a dome‐shaped, dark brown or black lesion that bleeds when it ulcerates. It is most found on the trunk of the body, though it can affect any portion of the body, even areas that are covered, like the axillae and buttocks. The irregular edge and frequent lack of different hues can cause a delay in detection. The lesion may have an irregular contour and vary in color. Compared to other melanomas, it often exhibits symmetry and a distinct border (Alasadi and Alsafy 2017).

#### 1.2.3 Acral Lentiginous Melanoma

This type of malignant melanoma is an extremely rare disorder. Normally, it begins unevenly and flatly, becomes raised, eventually becomes nodular, and occasionally has pronounced irregularities and a notched border. It typically manifests in an acral region or on a mucous membrane. The dimensions are at least 0.9 and up to 12 cm. Elevated, blue, black, or amelanotic nodules or papules that are frequently ulcerated are symptomatic of advanced lesions. It usually starts under the cuticle and leaves the nail plate with a pigmented streak (Alasadi and Alsafy 2017).

#### 1.2.4 Lentigo Malignant Melanoma

Lentigo is a particular type of malignant melanoma that primarily affects older people's faces and other sun‐exposed areas of their bodies. It manifests as various shades of pink, gray, blue, and white. The boundaries are exceedingly uneven and frequently marked with notches. The overall dimensions could range from 1.0 to 2.0 cm or more, it starts as a macule of tan that progressively spreads to the periphery and darkens in a variety of tones. After that, it becomes palpable and develops into a darkening papule, nodule, or plaque characteristic of melanoma (Cheung 1997).

### 1.3 Conventional Therapies

Melanoma is the most aggressive and deadliest type of skin cancer, even though it makes up only 1% of all skin malignant tumors (ACS 2017a, 2017b). The majority of those affected with this disease, regardless of gender, are Caucasian, and if it spreads to other areas, there is little chance of recovery (Jiang et al. 2016; Bombelli et al. 2014). For this reason, early detection of this malignancy is essential to the effectiveness of the patient's treatment. The clinical guidelines by the European society for medical oncology emphasize the need for a thorough diagnosis to determine the tumor stage. In certain cases, a mutation test is also necessary (Dummer et al. 2012). The US Food and Drug Administration (FDA) has approved several medicines in recent years. The possible treatment options for tumors include surgical resection, chemotherapy, radiation, immunotherapy, targeted therapy, and photodynamic therapy (PDT), depending on the tumor's characteristics (location, stage, and genetic profile). The main course of treatment for patients with stage i–iii melanoma is surgery (van Zeijl et al. 2021). The surgical techniques vary on the tumor's clinic‐pathologic characteristics. Adjuvant treatments, such as targeted therapy and immunotherapy are advised to increase survival.

#### 1.3.1 Surgery

The main treatment for localized melanoma is surgical excision of the tumor and surrounding healthy tissue; patients whose cancers are thinner than this but ulcerated or larger than 0.8 mm in thickness also undergo sentinel lymph node biopsy (Lee et al. 2013). The remaining lymph nodes in the region may occasionally be removed if melanoma cells are discovered in the sentinel lymph nodes. Metastatic tumors may also be surgically removed in certain circumstances; however, this is not always the case and further treatments such as radiation and chemotherapy can be used in such resilient tumors.

#### 1.3.2 Chemotherapy

Chemotherapy is a traditional cancer treatment that damages DNA in aberrant or uncontrollably dividing cells by using anticancer medications (Aung et al. 2017). A precise dosage of chemotherapy can cause cancer cells to become cytotoxic at the appropriate rates of apoptosis (Aung et al. 2017). However, the patient's condition of diagnosis determines how effective the treatment will be (Naidoo, Kruger, and Abrahamse 2018). Decarbonize and temozolomide are two medications that have been used in chemotherapy to treat metastatic melanoma, dacarbazine showed less than 5% of patients having a complete response, and 2%–6% of patients lived for 5 years (Kim et al. 2010) and less of an improvement in the median progression‐free survival with temozolomide.

#### 1.3.3 Radiation Therapy

In the United States, 1%–6% of melanoma patients receive radiation therapy. Radiation therapy is a medical intervention that targets cancerous cells by using high‐energy radiation to destroy them and inhibit their growth and division (Gianfaldoni et al. 2017). Genetic material is harmed by high radiation energy, which prevents the cells from dividing and multiplying further (Baskar et al. 2012). When utilized in an interdisciplinary therapy approach, radiation therapy can be combined with surgical excision to reduce local occurrence and enhance prognosis (Kim 2017).

#### 1.3.4 Targeted Therapy

Targeted therapy is a curative treatment that uses small‐molecular inhibitors or antibodies to block specific cancer genes, proteins, or the environment of the tumor that promotes cancer development and survival. To address the molecular flaws in melanoma, several targeted medicines have been created. The BRAF inhibitors, vemurafenib, and dabrafenib, which were licensed in 2011 and 2013, respectively, to treat metastatic and incurable BRAF‐mutated melanomas, are the most promising of these (Chapman et al. 2011; Rebecca, Sondak, and Smalley 2012). Nevertheless, the majority of patients experience secondary resistance in a comparatively short period these medications are very effective for about half of patients with BRAF‐mutated melanomas (Chapman et al. 2011; Rebecca, Sondak, and Smalley 2012; Scolyper, Long, and Thompson 2011). Researchers have been trying to create novel medications and drug combinations to have a more long‐lasting effect since some of the mechanisms by which these secondary resistance forms have been identified (Table 1).

**TABLE 1:** The different stages of melanoma and the typical conventional methods of therapy.

Stages	Conventional therapy	References
0	Surgery	(Mitchell, Karakousis, and Schuchter 2020; NCCN 2024)
I	Surgery	(NCCN 2024; J. A. Sosman 2023a)
II	Immune checkpoint inhibitor pembrolizumab and radiation therapy	(NCCN 2024; J. A. Sosman 2023b)
III	Adjuvant treatment with immune checkpoint inhibitors or with targeted therapy drugs, and radiation therapy	(NCCN 2024; Cohen and Tanabe 2023)
IV	Adjuvant therapy (Targeted therapy and Immunotherapy using drugs called checkpoint inhibitors)	(NCCN 2024; Cohen and Tanabe 2023)

#### 1.3.5 Immunotherapy

Molecularly targeted therapy targets CTLA‐4, which is overexpressed on activated T‐lymphocytes and functions as a negative regulator of T‐cell activation. Anti‐cytotoxic T‐lymphocyte antigens (CTLA‐4) antibodies are used in this approach (Zitvogel and Kroemer 2012). This strengthens the immune system's capability to eliminate cancerous cells. This immunotherapy targets CTLA‐4, programmed death ligand I or II death in metastatic melanoma cancer cells (Jazirehi, Lim, and Dinh 2016). Due to immune‐related adverse effects and resistance factors, this kind of therapy has the drawback that not all patients respond well to the entire course of treatment (Michot et al. 2016). Ipilimumab's efficacy as an anti‐ctla‐4 inhibitor in treating patients with metastatic melanoma was assessed in studies by Cirenajwis et al. (2015); however, serious adverse effects were reported. These modalities are briefly described under Table S1.

## 2 Advanced Treatment of Melanoma

### 2.1 Photodynamic Therapy

**FIGURE 1:**
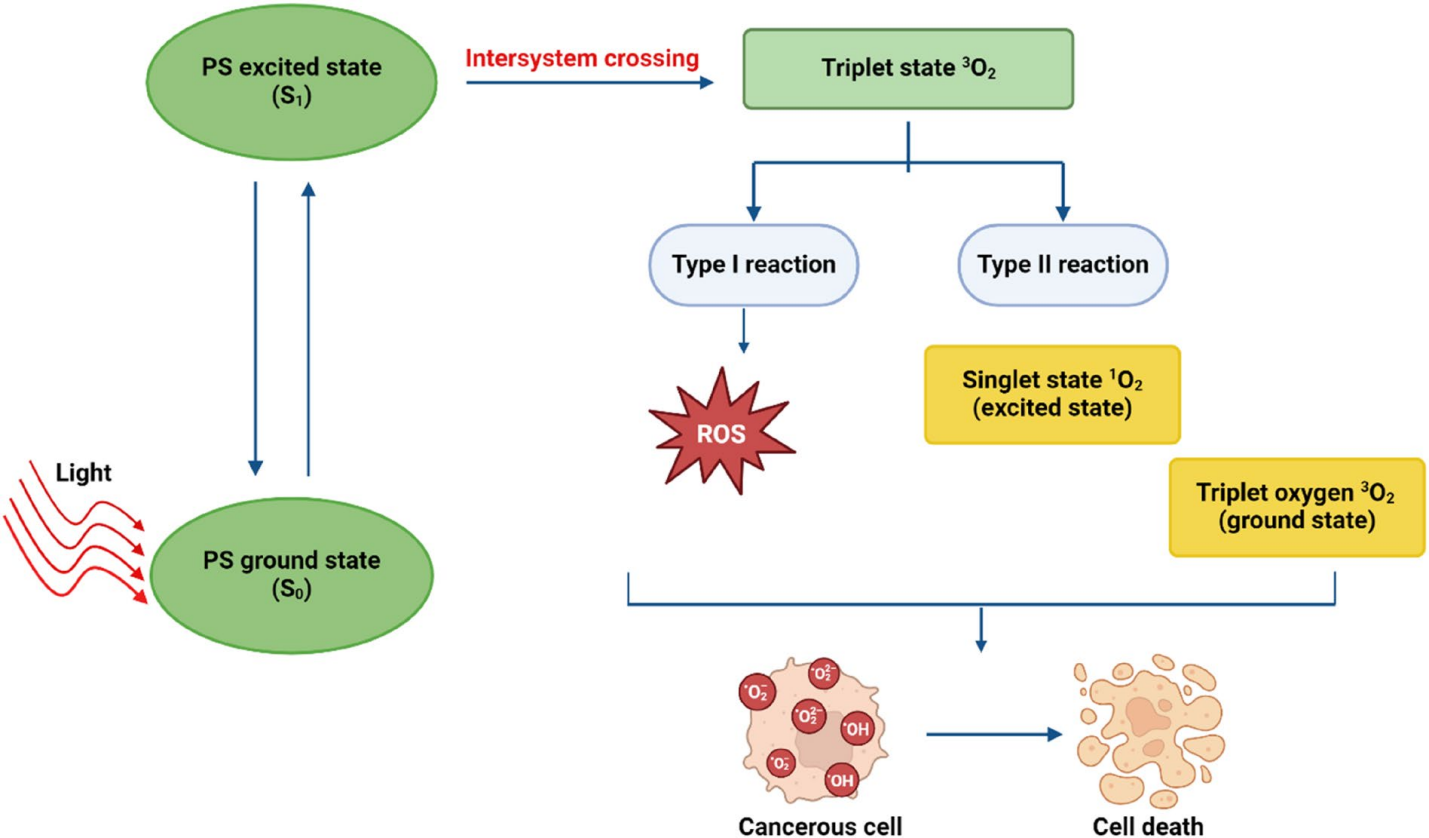
Mechanism of photodynamic therapy. A photosensitizer transitions from the singlet basic energy level S_0_ to the excited singlet state S_1_ when exposed to light at a wavelength that matches the photosensitizer absorption spectrum due to photon absorption. A photosensitizer molecule is propelled to its therapeutic triolet state by residual energy. Since PDT used light, oxygen, and photosensitizer, reactive oxygen species are created inside the biomolecules of cancer cells and cause cell death.

Photodynamic therapy (PDT) is an innovative, rapidly developing treatment for both cancer and non‐cancerous disorders, it combines light, oxygen molecules, and a photosensitizer (PS) (Kwiatkowski et al. 2018; Mansoori et al. 2019). When a PS is excited by a particular light wavelength, energy is transferred to the oxygen molecules or nearby biomolecules, resulting in the production of harmful Reactive Oxygen Species (ROS) that ultimately cause cell death (Figure 1). Compared to chemotherapy, radiation therapy, and surgery, PDT is a much superior treatment option because it is non‐invasive and eliminates cancer cells by passive PS build‐up without damaging healthy tissues (Hong, Choi, and Shim 2016; Sekhejane, Houreld, and Abrahamse 2014).

#### 2.1.1 Mechanism of Action of PDT

PDT causes cell death through the type I and type II photochemistry pathways, two different modes of action (Sekhejane, Houreld, and Abrahamse 2014). Both processes heavily depend on the oxygen molecules that are identified in cells. The process of photodynamic therapy utilizes a PS that once it enters the cell and is irradiated with light, emits light with a wavelength that matches the absorbance range of PS until it absorbs. The PS transitions from the excited singlet state S^1^ to the fundamental energy state S^0^. A PS molecule with residual energy travels to the right, therapeutic form of the chemical, the excited triplet state T_1_, where part of the energy radiates as the quantum of fluorescence.

**FIGURE 2:**
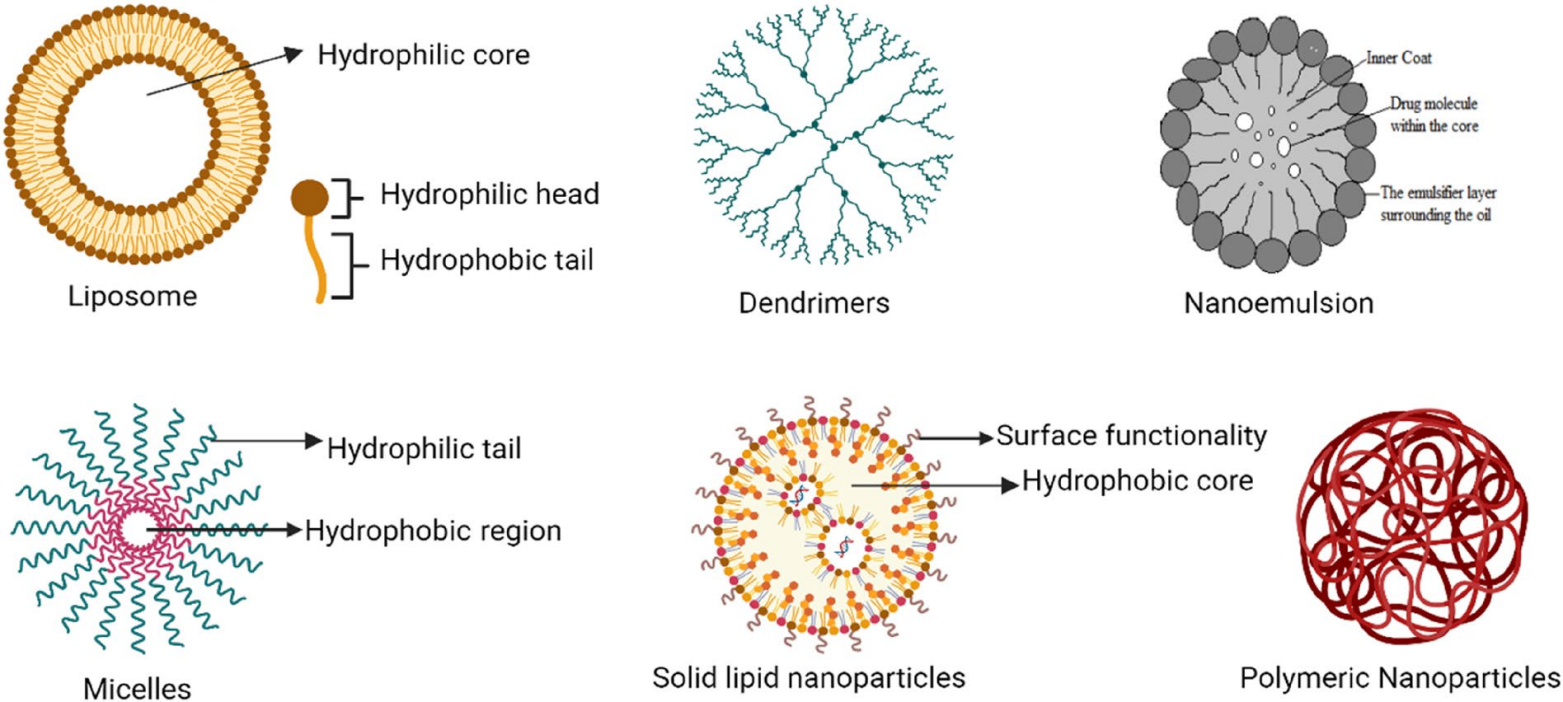
A schematic representation of the many nano‐formulations/nanocarriers utilized in drug administration, such as liposomes, dendrimers, polymeric nanoparticles, nano‐emulsion, and lipid solid nanoparticles.

During type I reactions, the excited triplet state of the PS transmits energy to neighboring proteins (Kwiatkowski et al. 2018). When an electron or a hydrogen atom is transferred between the PS and the substrate, free radicals are created (Hong, Choi, and Shim 2016). The resulting free radicals combine with oxygen to produce ROS, such as hydroxyl and superoxide radicals (Hong, Choi, and Shim 2016).

Type II reactions produce an extremely oxidizing singlet state of oxygen by directly transferring energy between the excited PS and the ground state of oxygen (Kwiatkowski et al. 2018; Hong, Choi, and Shim 2016). The resulting singlet oxygen species and ROS can damage proteins, lipids, and other biomolecules in the tissues targeted by the tumor, leading to either necrotic or apoptotic tumor cell death. While necrotic cell death is observed due to loss of cellular integrity, apoptotic cell death is typically triggered when cytotoxic ROS or singlet oxygen species generation damages cellular mitochondria. If it only impairs lysosomes or endoplasmic reticulum function, more autophagic forms of cell death are found (Kwiatkowski et al. 2018).

PDT involves activating a PS at a particular wavelength to induce excitation, as depicted in Figure 1. Two types of reactions take place in the excited state, also referred to as a triplet state. Superoxide anion radicals are created during type I reactions, and when these radicals interact with oxygen, oxygenated products are created. In type II reaction, the triplet excited state can transfer its energy directly to the oxygen, as a result producing singlet oxygen called reactive oxygen species (ROS).

#### 2.1.2 Potent PSs for Metastatic Melanoma PDT Therapy

Photosensitizers are produced chemically or naturally, and they are described as chromophores. A chromophore is a group of conjugated unsaturated bonds that absorb visible light at a particular visible wavelength and have a high molecular absorption coefficient. One of the most crucial steps in PDT is choosing the appropriate PS, which is essential for the most effective and fruitful course of treatment (Oniszczuk et al. 2016). Photosensitizers produce cytotoxic reactive oxygen species (ROS) that can physically and chemically destroy malignant tissues in which they are located. A PS that is suited for PDT applications should be a single, readily synthesized molecule with low cytotoxicity before particular spectrum light activation, high purity, and no known adverse effects.

**FIGURE 3:**
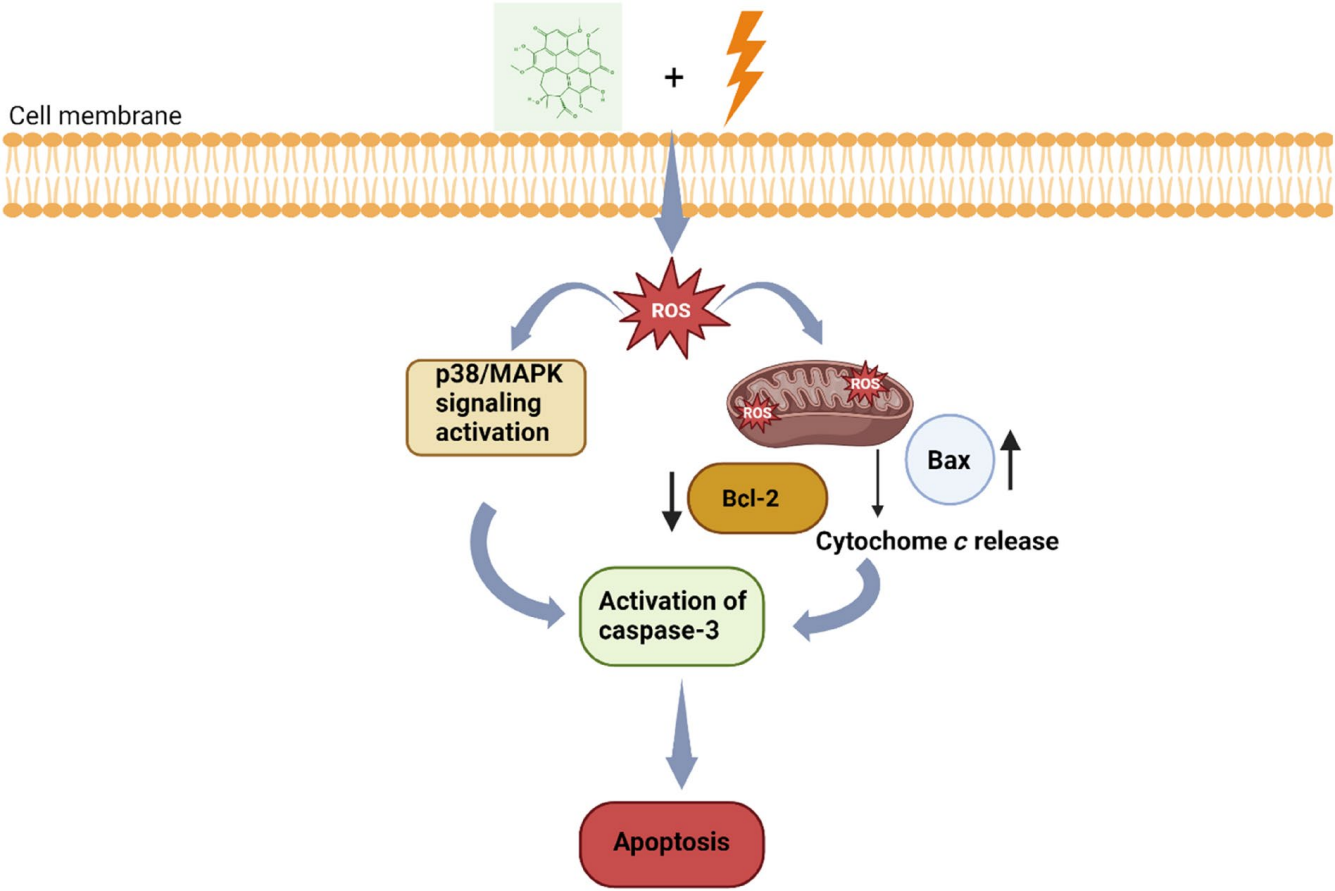
The figure shows how light induced reactive oxygen species are produced when hypocrellin is illuminated. These ROS then interact with biomolecules such as mitochondria, leading to activation of signal pathways as well as an imbalance in proapoptotic protein like Bax, Back‐2, and cytochrome *c*, ultimately causing the cell to undergo apoptosis.

Throughout the years, several PS classes have been studied for PDT therapy of metastatic melanoma (Vera et al. 2015). However, there are several elements to take into account when deciding which kind of PS to use with a specific PDT treatment, including the type of cell death the PS generates, its features, its method of action, and its localization (Atif, Zellweger, and Wagnieres 2016). Photosensitizers are usually less likely to cause mutations, DNA damage, and carcinogenesis since they reside in most cellular organelles other than the nucleus (Chen et al. 2017). Based on the photochemical and photophysical properties of PSs about their cellular mechanism of action, three generations of PSs are used in PDT applications (Josefsen and Boyle 2008). The localization of first‐generation PSs typically results in severe side effects and vascular tissue damage, suggesting that their exact localization within the cells is restricted (Yoon, Li, and Shim 2013). Second‐generation PS primarily causes cytotoxicity of tumor cells suggesting that PS is localized in organelles such as plasma membrane, endoplasmic reticulum, mitochondria, and lysosomes more passively (Abrahamse and Hamblin 2016). As a result, second‐generation PSs cause far fewer side effects than first‐generation PSs (Abrahamse and Hamblin 2016). Third‐generation PSs are photosynthetic medications that have additional targeting biomolecules added to them to improve the way the drug is specifically absorbed and adsorbed by cells (Kataoka et al. 2017).

Porphyrins, phthalocyanines, chlorins, and porphycenes are the four primary groups of photo‐chemicals (PSs). Because porphyrins are relatively stable and belong to the first generation, they tend to generate photosensitivity and have poor tissue penetration depth. Despite this, porphyrins have been employed frequently in PDT applications (S. Singh et al. 2015). Chlorins are second‐generation polyphenols (PSs) derived from derivatives of chlorophyll or porphyrin. Chlorins have a high PDT efficacy rate when treating squamous and basal cell carcinomas, according to reports by Jerjes, Hamdoon, and Hopper (2017). Second‐generation PSs called phthalocyanines offer even higher PDT efficacy because they have a diamagnetic metal ion that permits deep tissue penetration by laser light with much fewer phototoxic side effects (Jiang, Shao, et al. 2014) (Table 2). Reports on the current PSs that have been used in PDT for melanoma treatment. The photosensitizers used in cancer have proven that they ultimately eradicate cancerous cells when illuminated with light of a suitable wavelength. These have shown effective results of about 60%–70% cytotoxic induction of apoptotic cell death in cancerous cells.

**TABLE 2:** Review of photosensitizers used in photodynamic therapy for melanoma.

Photosensitizers (PS)	Cells	Results	References
Phthalocyanine	Achromic melanoma cells	In cultivated cells, substantial amount of photokilling was detected.	(Schmitt et al. 2006)
Metallophthacyanine (MPc) and 5‐aminolevulinic acid (5‐ALA)	Human metastatic melanoma (A375)	There have been reports of notable reductions in cell viability, ranging from 60% to 80%, along with the cytotoxic induction of apoptotic cell death.	(Robertson, Abrahamse, and Evans 2010)
Ruthenium porphyrins	Human melanoma cells	There was a noticeable significant reduction in cell viability, ranging from 60% to 80%. When exposed to light, it appears to exhibit no phototoxicity but displays a greater degree of cytotoxicity in the dark.	(Sweigert et al. 2012)
5‐Aminolevulinic acid (5‐ALA)	Mouse melanoma cells (Mel25)	PS performed well in vitro at killing cells, but when tested in vivo, it had only modest effects.	(Córdoba et al. 2005)
Halogenated porphyrins	Human melanoma cells (A375)	Since P‐gylcoproteins have a far higher phototoxicity than Photofrin due to halogenated structure interfering with it, PD demonstrated a 30‐fold improvement in killing effectiveness when compared to Photofrin.	(Serra et al. 2010)
Verteporfin	Melanoma tumors in mice	There were noticeable reductions in tumor development as well as large necrotic patches in the tumor. Verteporfin's photosensitizer is dose‐dependent, with greater doses producing longer photosensitivity.	(Swavey and Tr 2013)
Bacteriochlorin	Mouse melanoma cells (S91)	After 24 h of treatment with vascular‐targeted PDT, S92 cells were killed. Tumor regrowth and significant Po_2_ compensating effects were the results of cellular targeted PDT.	(Davids et al. 2013)

Photodynamic therapy has been used successfully to treat basal cell carcinoma, head, and neck tumors, which are overexpressed and thus easily irradiated by laser light. Internally metastasized skin malignancies, on the other hand, are much more difficult to cure with PDT because they are exposed to significantly less laser light irradiation (Yu 2016). Furthermore, metastatic melanomas are pigmented with melanin, which prevents efficient laser light from reaching the target site, thus, PDT treatment for skin cancers and tumors that have metastasized is frequently less effective (Slominski, Zmijewski, and Slominski 2015). To address these problems, recent research efforts have concentrated on creating treatment modalities that will target the tumors and have a much higher wavelength with deeper tissue penetration and enhance ROS generation, in addition to much more compacted lasers that can deliver light endodermally (Zhou et al. 2016). One of the treatment modalities that is effective on metastatic melanoma is photoimmunotherapy. The medical treatment known as photoimmunotherapy involves the formation of photo immunoconjugates by coupling particular antibodies with photosensitizers (Kobayashi and Choyke 2019; Kobayashi et al. 2021). The treatment combines the benefits of conventional PDT with the capacity of antibodies to target specific targets precisely (Mitsunaga et al. 2011). It is well established that this therapy has enormous potential for tumor diagnosis, treatment, and prevention. Tumor photodynamic therapy in combination with immunotherapy has the potential to improve the immune system, which can be beneficial for the treatment of metastatic cancer. Tumor antigen sources can be effectively produced by photo‐induced cell apoptosis, autophagy (Glick, Barth, and Macleod 2010; Kessel, Vicente, and Reiners 2006), or necrosis (Van Straten et al. 2017). Therefore, combining immunotherapy and phototherapy stimulates the immune system against tumors by exposing it to tumor antigens. Photoimmunotherapy has the potential to be used to treat metastases as well as residual tumors. Photoimmunotherapy, in contrast to other conventional therapies, stimulates a particular antitumor immune response rather than weakening the host antitumor immune response. It attracts dendritic cells by inducing immunogenic cell death (IDC) and quickly releasing tumor‐specific antigens and danger signals related to membrane damage. After migrating to the tumor site, DCs deliver antigens unique to the tumor, trigger the proliferation of tumor‐specific T lymphocytes, and promote the death of the tumor cells (Liu et al. 2021).

### 2.2 Natural Photosensitizer

Plants and other living organisms can produce a variety of naturally occurring compounds that act as PSs by absorbing UV‐A or white light. Diversity cannot be regulated because there are still a lot of unidentified natural PS compounds. Hypericin and curcumin are two natural compounds that have been extensively studied as one of the most effective PSs over the years. *Hypericum perforatum*, sometimes known as John's wort, is a flowering plant that heals burns and other various skin conditions. This plant also has antiviral, antidepressant, antibacterial, and anticancer activities, according to clinical study. However, the precise processes or mechanism behind these effects remain poorly known (Kubin et al. 2005).

#### 2.2.1 Use of Various Natural PS for PDT of Cancer

Since ancient times, a variety of human illnesses have been treated using herbal medicine extracts, and natural products (Mohammadi et al. 2016). It is obvious that natural products should be further searched as potentially useful agents in cancer therapy, as most half of currently available medications are derived from various types of plants (Mohammadi et al. 2016). Combining PDT with therapeutic modalities, such as immunotherapy, chemotherapy, radiation, or even herbal medicine, may be a potential strategy against a variety of cancer types, as the outcomes of monotherapy have been very encouraging (Moreira et al. 2008). It is important to also note that combination therapy can more successfully stop the growth of cancer cells and generally has less adverse effects than single agent therapies. Combination treatment may occasionally boost response by increasing the anticancer chemotherapeutic medicines' absorption into cancer cells and tumors. Therefore, PDT combination treatment might be an effective method for treating cancers that are resistant to drugs (Khdair et al. 2010).

Herbal medicine has been utilized since ancient times, as was previously noted. *Hypericum perforatum* (HP) is one of the most well‐known herbs used in PDT (Skalkos et al. 2006). One isolated HP molecule that may be used as a first‐choice PS in PDT is called hypericin. Research has shown that the phototoxic impact of hypericin may be enhanced when combined with other PSs, such as chlorin (e6), and that both can be activated by light. Additionally, physical methods, such as causing hypothermia, can also be employed. It is demonstrated that the primary mechanism causing apoptosis in the mitochondria route involving caspase‐3 and caspase‐9, and that the inhibition of PI3K/Akt pathway mediated by vascular endothelial growth factor‐A inhibits cell proliferation (Skalkos et al. 2006). According to another study, when hypericin is activated with the right wavelength of light (600 nm), can kill cancer cells in vitro and tumors in vivo. The polar methanolic fraction of HP was shown in one in vitro investigation to have proapoptotic and antiproliferative properties in human bladder cancer (Kamuhabwa et al. 2001). Berberine is one of the isoquinoline derivative alkaloid isolated from *Rhizoma coptidis* and *Cortex phellodendri and* this photosensitizer has high fluorescence characteristics that were isolated from Chinese medicinal herbs, in a study presented by Liao Jing et al. (Liao, Pp, and Wu 1997). It was demonstrated that the utilization of those herbal extracts in conjunction with light significantly lower metabolic cell viability and promotes cell death. Active ingredients or fractions isolated from traditional Chinese medicinal herbs, including, *Rabdosia rubesens*, *Cortex magnolia officinalis*, *Rhizoma bupleuri*, and *Rhizoma polygoni cuspidate*, have been reported to have anticancer properties when combined with light activation (Wu, Luo, and Wang 2012). This research's finding implies that photosensitizing substances that have been separated from native plants may be used as an alternative in PDT. These substances include aloe‐emodin, hypericin, hypocrellin, cercosporin, thiophenes, anthraquinones, and tolyporphin (Table 3).

**TABLE 3:** Natural photosensitizers used in cancer treatment.

Natural photosensitizers	Indications	Cellular targets	References
Curcumin	Cytotoxicity against neural progenitor cells, anti‐inflammatory, anticancer, and antioxidants properties	Membrane of the lysosome	(Koon et al. 2006)
Chlorophyllin	Skin, breast, and bladder cancer	Mitochondria and lysosomes	(Du et al. 2014; Gomaa et al. 2012; B. Li et al. 2012)
Hypecerin	Nasopharyngeal carcinoma cells and bladder cancer	Membrane of the Golgi complex, mitochondria, endoplasmic reticulum (ER), and nuclear	(B. Li et al. 2012; Schempp et al. 1999; Vantieghem et al. 2001; Vantieghem et al. 1998; Weller et al. 1997; Zupkó et al. 2001)
Hypocrellin	Skin conditions, and cervical cancer	Mitochondria and the lysosomal compartment	(Miller et al. 1997; D. Zhenjun and Lown 1990)
Thiophenes	Effects of cytotoxicity on human cancers, including cancers of the skin and cervix	Lysosomal compartment	(Marles et al. 1992; Y. Wang et al. 2007)
Tolyporphin	Metastasis	Particular vesicles and the perinuclear area	(Morlière et al. 1998)

### 2.3 Hypocrellin‐Mediated PDT in Cancer/Melanoma

Hypocrellins are derived from the parasitic fungus *Hypocrella bambusae* and Shirai, which are primarily found in Asia. Members of the large class of perylene quinonoid pigments are hypocrellin A and B (Zhenjun and Lown 1990). These compounds used in PDT as photosensitizers have been thoroughly investigated. Hypocrellins have high singlet oxygen quantum efficiency, and this has drawn interest in it being a possible PS for PDT. Reactive oxygen species (ROS) and hypocrellin radicals are produced when the chemical is activated, and these may increase the phototoxicity of cells. Hypocrellin has the capability to be chemically altered simply to increase phototoxicity, pharmaceutical kinetics, dissolution, and red‐light absorption (Jin et al. 2013). Other advantages of these specific photosensitizers over others are their high light toxicity and low toxicity in the darkness, ease of synthesis and purification, high triplet quantum yield, and quick removal from healthy tissues. The substance hypocrellin can be found in lysosomes, mitochondria, and membranes of cells and has an affinity for lipids. (Jiang, Leung, et al. 2014). Through apoptosis and necrosis, which is caused by lipid membrane peroxidation, cells die (Jiang, Leung, et al. 2014). Hypocrellins have demonstrated efficacy in cancer phototherapy encompassing skin and breast cancer treatment (Jiang, Leung, et al. 2014). They also exhibit antiviral and antitumor characteristics when activated by light (Jiang et al. 2013).

The Chinese herb *Hypocrella bambusae* is regarded as an effective photosensitizer (Jiang, Leung, et al. 2014). According to studies by Hudson and associates, hypocrellin B‐mediated photodynamic action has been proven to effectively kill tumor cells and dangerous bacteria. Hypocrellin B has been found to possess significant physicochemical advantages over hematoporphyrin derivatives (HpD). These advantages include the presence of a compound, a defined chemical structure, high singlet quantum yield and dual photochemical mechanisms of type I and type II (Jiang, Leung, et al. 2014). Research has indicated that hypocrellin B exhibited a more potent photodynamic killing effect on tumor cells. However, the visible wavelength range of hypocrellin B's greatest absorption spectrum lies between 460 and 470 nm (Jiang, Shao, et al. 2014). Researchers have utilized a cutting‐edge blue light source made of light‐emitting diode (LED) with a wavelength of 470 nm in order to efficiently activate hypocrellin B. Their earlier research demonstrated that blue light from LED source might activate hypocrellin B leading to death of cancer cells (Jiang, Leung, et al. 2012).

Several studies suggest that different derivatives of hypocrellin have been utilized in PDT to treat a wide range of illnesses, including bacteria (*Staphylococcus aureus*), lung, ovarian, and upper respiratory tract cancer (Mansoori et al. 2019) Hypocrellin B has been demonstrated to exhibit antiviral, antibacterial, antifungal, and anticancer properties in a number of investigations (Jiang, Xia, et al. 2012). Remarkably, when exposed to visible light, the compound produces large amounts of ROS. The viability of *S. aureus* significantly decreased following photodynamic therapy of hypocrellin B, according to a study done to investigate the impact of hypocrellin on *S. aureus*. Bacterial cells exposed to hypocrellin B and PDT also showed remarkable ultrastructural damage. These results suggested that *S. aureus* might completely be eradicated by PDT combined with hypocrellin B, however more clarification regarding the underlying mechanism needed to be understood (Jiang et al. 2013).

The impact of hypocrellin B‐photodynamic treatment (HB‐PDT) on the lung cancer cell line A549 was documented by Zhou in 2004. It caused apoptosis, and HB‐PDT'S IC_50_ was 33.82 ng/mL (Zhou 2004). A high IC_50_ value of 34.16 mg/mL was reported by Shang et al. in 2005 to suggest that HB‐PDT could cause esophageal cancer cells to undergo apoptosis (Shang et al. 2005). The apoptosis of breast cancer cells with HB‐PDT was examined by Y. Jiang et al. It slowed the proliferation of breast cancer cells and down regulated the expression of the HER2 gene (Jiang, Xia, et al. 2012). In 2014, Y. Jiang et al. discovered the impact of HB‐PDT on cancer cell adhesion, migration, and death in vitro. They also discovered that HB‐PDT prevented human ovarian cancer HO‐8910 cells from adhering to one another and from migrating, and that it raised the early apoptotic and late apoptotic (necrotic) rates to 16.40% and 24.67% respectively (Jiang, Leung, et al. 2014). These findings demonstrated that the activated reactive oxygen species (ROS) responsible for cell death brought on by the photosensitization of hypocrellins were singlet oxygen (^1^O_2_) and superoxide (O^−2^) (Weishaupt, Gomer, and Dougherty 1976). Furthermore, there has not been much research on the effects of HB‐PDT treatment on melanoma; for this reason, emerging studies are aimed at examining how HB‐PDT affects the A375 melanoma cell line.

#### 2.3.1 Limitations and Approaches to Enhance Hypocrellin‐Mediated PDT

Hypocrellin is a naturally occurring photosensitizer with exceptional photodynamic properties. Additionally, the antiviral and anticancer activities of the luminous body as well as its clearance speed can be increased by this strategy (He, An, and Jiang 1999a; He, An, and Jiang 1999b). Combining hypocrellin with phototherapy to target the ideal infrared area for photodynamic therapy has been shown to be a successful way to treat various skin diseases and malignancies. Research has also shown that hypocrellin can be involved in both type I and type II photodynamic reactions. This means that in addition to active oxygen species (O_2_ and O^−2^), hypocrellin radicals can also contribute to the photodynamic damage of cells (An, Hu, and Jiang 1996; He, An, and Jiang 1999a; He, An, and Jiang 1999b). In particular, hypocrellin radicals have been identified as important intermediaries in light‐induced cytotoxic reactions because of their capacity to produce toxic materials. It is commonly recognized that the optimal wavelength range for cancers to be treated with phototherapy is between 600 and 900 nm (Henderson and Dougherty 1992; Hu and Jiang 1996; He, Jy, and Lj 1998). However, a significant drawback of hypocrellin is that they only absorb light energy at wavelengths shorter than 600 nm. Many attempts have been made to shift the absorption wavelength toward the infrared phototherapeutic window by changing the chemical structure (He, An, and Jiang 1999a, He, An, and Jiang 1999b). Two photon excitation techniques can successfully tackle this problem without altering the chemical structure of the photosensitizer that is currently in place. In addition, hypocrellin are lipophilic chemical compounds that inhibit medication delivery and bioavailability while promoting cellular absorption (Engelhardt et al. 2011; Ishikawa and Hashimoto 2011). Moreover, most recent research data have demonstrated that the use of nano‐formulations in conjunction with hypocrellin B can greatly increase its solubility, bioavailability, and photodynamic efficacy (Gomes, Lunardi, and Tedesco 2007). To effectively use hypocrellin in cancer therapy the use of nanostructures as carriers and chemical m Ishikawa odification were implemented to produce HB with longer wavelength. 1,2‐Diamino‐2‐methyl propane was used to modify HB to create DPAHB, which has a broad absorption range of up to 800 nm. Poly(ethylene glycol)‐b‐poly(lactic‐co‐glycolic acid) (PEG‐PLGA) was loaded. Under CLSM monitoring, DPAHB coated with nanoparticles occurred to have built up in the tumor 12 h after administration. This demonstrates the improvement of tumor targeting and the window of opportunity for tumor treatment (Zheng et al. 2018). Furthermore, protein nanocages have also been shown to be more superior transporters of PSs due to their biocompatibility and biodegradability. Proteins like Apoferrin (ATF) have been utilized extensively for targeting tumor and for deeper penetration of tissues because ATF is recognized by overexpressed Transferrin receptor 1 (TfR1) and Chemokine (C‐X‐C motif) Receptor 4 (CXCR4) on the surface of tumor cells. HB‐ATF NPs are therefore able to enter the cell more quickly via CLSM than they would have if the ATF nanocages had not been coated (Jiang et al. 2017). The modification of HB does not only assist in increasing the absorption of the photosensitiser by cells, but it also assists in greater production of ROS levels and that improves its use in PDT.

### 2.4 Hypocrellin Nano‐Formulation to Enhance PDT Efficiency

Current phytochemicals that are utilized as medications have high molecular weights, are extremely water soluble, and have poor absorption qualities because they cannot pass through lipid membranes (Shakeri and Sahebkar 2016). By contrast, poorly water‐soluble medicines with a sluggish rate of drug absorption make up over 40% of new chemical entities (Savjani, Gajjar, and Savjani 2012; Sharma et al. 2009). This reduces their therapeutic usefulness since both lead to reduced bioavailability and ineffective medication delivery (Saraf 2010). One of the main causes of inadequate absorption of conventional medications is the difficulty in determining the ideal formulation that takes into consideration the drug's physiochemical characteristics as well as the kind of target site and disease (Gupta, Kesarla, and Omri 2013). To increase the therapeutic value of pharmaceuticals, there has been a surge in interest in the development of nano‐formulated medications in recent years (Kumari, Yadav, and Yadav 2010). These medications' high surface to mass ratio and quantum size effects, such as electron confinement during absorption and drug delivery, are distinctive qualities of the nano form (de Jong and Borm 2008). These characteristics are essential for overcoming the problems of phytochemicals limited therapeutic absorption when used as medications or novel chemical entities. Drug delivery research has used a variety of nano‐formulations to reduce side effects, including toxicity risks, and to enhance targeted drug delivery, bioavailability, solubility, and drug retention duration (Knight 1981; Devalapally et al. 2006; Kreuter 2014).

In nano‐formulations, the drugs are attached to the drug carrier, dissolved, entrapped, or encapsulated. Their usual size is between 10 and 100 nm (Soppimath et al. 2001; Dowling 2004). A few important factors need to be carefully considered when formulating nano‐drugs. The formulation needs to facilitate the drug's transportation from the site of administration to the site of action while also shielding it from the negative effects of pH, enzyme harm, and potential biochemical breakdown. Additionally, the formulation needs to facilitate the administration of lower quantities of the drug in order to produce a strong pharmacological effect and release the payload in its active form in and around the target site (Devarajan and Jain 2015). Pharmaceutical industries use a variety of nano‐formulations, including liposomes, polymers, dendrimers, nano‐emulsions, micelles, for drug delivery. Dendrimers are synthetic, hyperbranched polymeric macromolecules that have a well‐defined core, backbone, and multivalent periphery to form globular tree‐like structures (Svenson 2009; Svenson and Tomalia 2012). These dendrimers carry a variety of drugs by encapsulating them within their cores (Lee et al. 2005), or by using covalent conjugations. They can also be functionalized to suit the requirements of the delivery site, which makes them a better option for creating multifunctional drugs (Wolinsky and Grinstaff 2008). Liposomes are lipid bilayers composed of synthetic or natural phospholipids in an aqueous phase that encapsulate drugs in a closed spherical vesicle (Malam, Loizidou, and Seifalian 2009). Liposomes are highly stable and thus useful as drug delivery carriers (Rao and Geckeler 2011; Okada and Toguchi 1995). Polymeric nanoparticles, such as nanospheres and nano‐capsules, enhance the water solubility of drugs (Wu, Luo, and Wang 2012) while also controlling the drug release rate (Li et al. 2012; Gomes, Moreira, and Castell‐Perez 2011; Keawchaoon and Yoksan 2011; Hosseini et al. 2013). They are very stable and thus valuable ad drug delivery vehicles for steroids, vaccines, and genetic materials (Gregoriadis and Florence 1993). Nano‐emulsions have droplet‐sized emulsions ranging from 20 to 200 nm, making them highly robust against sedimentation (Sonneville‐Aubrun, Simonnet, and L'alloret 2004; Solans et al. 2005). These emulsions have fascinating size‐dependent features, such as optical transparency (Mason et al. 2006) and good shelf stability against gravity‐driven particle creaming (Russel et al. 1991), which can help improve medication efficacy in the pharmaceutical industry (Solans et al. 2005). Micelles are amphiphilic molecules with a core‐shell configuration (Jeetah, Bhaw‐Luximon, and Jhurry 2014). The inner hydrophobic core (Letchford and Burt 2007) and outer hydrophilic corona (Vonarbourg et al. 2006) make them excellent drug carriers, allowing for sustained circulation in biological systems (Jones and Leroux 1999; Kwon et al. 1993; Figure 2).

Moreover, these nano‐formulations (Figure S4) can either be delivered passively or actively for efficient PDT. Passive PS absorption occurs when the medication accumulates in tumor cells due to nano‐formulation composition and size, with overall drug uptake influenced only by the surrounding tumor environment (such as hypoxia or low pH) and the EPR effect (Pellosi, De Jesus, and Tedesco 2017). In active absorption, the PS medication is delivered to a specific target tumor site via molecular recognition mechanism (Naves et al. 2016). The nano‐formulations are functionalized with target molecules that precisely bind to receptors overexpressed in tumor cells resulting in increased PDT medication absorption (Nicolas et al. 2013). However, Maeda (2012) found that PDT PS carrying nano‐formulations that use passive targeting strategy have a greater impact on healthy surrounding tissues than active targeting strategies, because passively absorbed NP drugs cannot distinguish between cancerous and normal cells and thus occasionally distribute in healthy tissues. To improve tumor PS drug accumulation selectivity and prevent undesired side effects, recent research has focused on developing nano‐formulations PS bioconjugates with specifically targeted action for PDT cancer applications (Pelaz et al. 2017).

#### 2.4.1 Synthetic Hypocrellin Nanoparticles With Biomaterials

They are known as nanospheres or nano‐capsules, are nanoparticles, which transport medications through covalent bonding, chemical adsorption, or physical adsorption. In the past, 100 nm sized hypocrellin nanoparticles were made using polysaccharides or gelatins (Zhao, Zhao, and Xie 2005; Zhao, Xie, and Zhao 2004; Zhao, An, and Xie 2004). In addition to being a drug delivery system, the preparation accounts for the majority of PDT activity. Numerous varieties of hypocrellin nanoparticles have been described up to this point, such as lipid‐coated gold nanocages, pH responsive silica nanoparticles, hierarchical gold/copolymer nanostructures, and nanoscale porous ceramic particles (Zhou et al. 2009; Zhou et al. 2011; Li et al. 2010a; Li et al. 2010b; Paramaguru et al. 2011; Gao et al. 2012). Because certain hardeners are included in the formulations, they are typically quite stable. However, PDT of microvascular illnesses is not a good candidate for controlled release medications, whereas PDT of solid tumors may be.

#### 2.4.2 Hypocrellin‐Metal Ion Complexes

Hypocrellins easily form water‐soluble complexes with ions of aluminum (III) magnesium (II), zinc (II), or lanthanum (III) (Zhou et al. 2005). Hypocrellin metal ion complexes have been shown to improve tumor water solubility and absorption within the phototherapeutic window (600–900 nm) (Ma, Zhao, and Jiang 2001; Zhao, Zhao, and Xie 2005). The complex's molecular weight was generally unknown, possibly as a result of the polymer‐like structures that formed (Ma, Zhao, and Jiang 2001). Certain Cu (II), Co (III), and Oxovanadium (IV) HB complexes have been observed to cause more effective photodamage to DNA than free HB. Hypocrellin has been shown to produce strong ligand bonds when interacting with thiols, allowing the compounds' reactive groups to bind to nano‐formulations. According to the chemical structure of HB, two types of locations are likely to be reactive with thiol compounds: the aromatic ring (positions 5 and 8), and the side ring (position 13). As expected, the aromatic ring is significantly more reactive than the side ring (He, Jy, and Lj 1998). This thus, implies that the nano‐formulations are likely to be thiolated before their reaction with hypocrellin B, which facilitates the strong link between the two.

#### 2.4.3 Liposomal Hypocrellins

Lipid bilayers that either spontaneously form or are artificially built by phospholipids make up the liposome. The liposome is a frequently utilized drug delivery system of lipophilic medications because of its superior biocompatibility, targeting and regulated drug efflux rates. One example of liposomal preparation is verteporfin, a PDT treatment for AMD that is used in clinical settings (Ebrahim, Peyman, and Lee 2005). The liposome of HA and HB, which can preserve around 70% of PDT activity of HA and HB was previously prepared using an ultrasonic technique (Yu et al. 2001; Zhang et al. 2009). The liposomal HB photosensitization generated singlet oxygen, hydroxyl radicals, indicating the involvement of type I and type II processes indicated in Figure 1. (Yu et al. 2001). By delivering active pharmaceuticals to their intended tissues, liposomes lessen peripheral drug toxicity and increase the therapeutic potential cargos. The effectiveness of some alternate tactics based on magnetic and light fields was also increased.

#### 2.4.4 Hypocrellin Nano‐Micelles

Surfactant molecules are used to load lipophilic medicines into a micelle. The particle diameters of HA and HB triton X‐100 micelles were produced to be between 5 and 100 nm (Zhang, Zhang, and Zhang 1998; Zhao, Xie, and Zhao 2004; Zhao, An, and Xie 2004; An et al. 2003; Liu et al. 2000). Triton X‐100 HB micelles were found to be able to sustain the majority of the photosensitization activity. Due to its tiny size, high drug loading, extended blood circulation, and selective tumor accumulation, the polymeric micelle is one of the most effective drug loading vehicles.

#### 2.4.5 Nano‐Emulsions

Some photosensitizers, such as aluminum phthalocyanine chloride (APIc), have the potential to be used in PDT application is restricted by their hydrophobicity. In order to enhance the pharmacokinetic impact and stop AIPc from aggregating in aqueous media, Muhelmann and associated created their nano‐emulsions by combining castor oil with cremophor ELP. In comparison to free AIPc, the in vitro findings indicated that the AIPc nano‐emulsion has strong photodynamic activity in human breast cancer MCF‐7 cells (Muehlmann et al. 2015). Therefore, the use of nano‐emulsion in antitumor therapy may be an effective way to increase the photodynamic activity of hydrophobic photosensitizers more so hypocrellins included. Similar to this, Zhang and colleagues added 5‐ALA to two different kinds of emulsions in order to increase the substances skin penetration.

#### 2.4.6 Dendrimers

A number of arms that emerge from the center of the dendrimer are made up of sugar, nucleotides, and amino acids (Din et al. 2017). Dendrimers are useful for cancer treatment because they reduce the amount of bioavailable metal in tumors. Since the traditional dendrimer synthesis technique is challenging, an efficient approach was developed to create poly (acylthiourea) and polythiourea dendrimers. To create one dendrimer generation in 4 h, this approach uses thiol‐methacrylate Michael addition process and isothiocyanate‐amine coupling (Moradian and Rahbarizadeh 2021). Numerous investigations have been conducted about the potential benefits of using dendrimers in medication delivery.

In summary, hypocrellin has been successfully incorporated into nanomaterials via conjugation, loading, and encapsulation; these bioconjugates aid in the uptake of the photosensitizer, delivery to the target site, and improve the absorption spectrum since hypocrellin B has lower absorption spectrum and that is normally its disadvantage in PDT. Photosensitizers are typically conjugated/encapsulated with co polymers such as Poly (lactic‐co‐glycolic) acid (PLGA) and polyethylene glycol (PEG), which are commonly utilized in drug delivery systems to encapsulate both hydrophilic and hydrophobic drugs. The US Food and Drug Administration (FDA) has approved these synthetic polymers, which have been utilized to encapsulate all types of anticancer drugs in nano‐formulations and they have good biodegradability, minimum toxicity, and high bioavailability (Khan et al. 2016). Hypocrellin has been integrated into nano‐formulations such as nano silver‐loaded polymeric nanoparticles, and HB‐NPs were synthesized using nanoprecipitation process under light protection (Gan and Feng 2010). Briefly, a known amount of HB and PLGA (ester terminated form with inherent viscosity) were dissolved in acetone. Nano silver was distributed in an aqueous phase containing alpha‐tacopheryl polyethylene glycol and stirred before the organic phase was injected. Another method is emulsion‐solvent evaporation it has been used to prepare hypocrellin‐loaded micelles. To create a water‐in‐oil (W/O) emulsion, add PEG, PLGA, and drugs (hypocrellin) to an organic solvent (oil phase). To create a W/O/W emulsion, the resulting emulsion was mixed with water and homogenized using sonication (Chen et al. 2019; Shen and TanTai 2020). Drug‐loaded PEG‐PLGA NPs were obtained by evaporating the organic solvent and filtrating them. This strategy, like the O/W single emulsion‐solvent evaporation method, encapsulates proteins and hydrophilic medicines, limiting their diffusion out of the NPs and enhancing entrapment efficiency and sustained release (Zhang et al. 2014). The use of PEG‐PLGA in nanoparticles formation and drug delivery is biocompatible and non‐immunogenic and it enhances the solubility, stability, and safety of anticancer drugs used to treat various types of cancer. Examples of the application of drug‐loaded PEG PLGA nano‐formulations in cancer treatment include the efficient use of several drugs loaded with PEG PLGA NPs and the results of the bioconjugates show improved drug encapsulation, controlled release, and efficacy against solid carcinomas. Reduced side effects while maintaining sustained release and anticancer characteristics in vitro and in vivo. Co‐loaded NPs, therefore, shows superior sustained release, targetability, and tumor growth suppression compared to single drug‐loaded NPs. These finding suggest that PEG‐PLGA NPs can effectively treat malignancies and reduce the need for frequent medication delivery (Zhang et al. 2022). The data presented show that PEG‐PLGA NPs have excellent potential and are widely used for controlled drug release and targeted delivery. To my best knowledge, no PEG‐PLGA NPs formulations have reached the worldwide market, despite its tremendous success in preclinical research. Only a few PEG‐PLGA based microparticles depot formulations were developed. A number of unique and general restrictions and challenges hinder PEG‐PLGA NPs ability to reach clinics. Low drug‐loading capacity and the initial burst release of pharmaceuticals are critical limitations that must be addressed (Danhier et al. 2012).

### 2.5 Hypocrellin Nano‐Formulation Therapy Targeting Oxidative Stress and Molecular Pathways in Melanoma

The effects of hypocrellin nano‐formulations therapy that precisely target oxidative stress and molecular pathways in melanoma have not received much attention. However, a novel treatment for a variety of malignancies employs hypocrellin and nano‐formulations. Reactive oxygen species (ROS) have a variety of functions in the metastasis, and survival of cancer. While low ROS production encourages cell growth, high ROS production can cause cancer cells to undergo apoptosis (Krammer and Verwanger 2012). The natural substance generated from plants, hypocrellin B, has a high singlet oxygen (_1_O^2^) quantum yield (Choi et al. 2019). A study on the photodynamic therapy of hepatocellular carcinoma using neutrophil membrane‐coated hypocrellin B nanoparticles (NM‐HB NPs) revealed a favorable response in the cells through JUNB/ROS signaling (Zhang et al. 2021). The outcome of this combination served as evidence that NM‐HB NPs stimulated by laser have a significant level of ROS products. Even though the pattern of how NM‐HB NPs affect cell apoptosis appears to be evident, it is further investigated to identify the function that ROS generation plays in the degradation of cancer. Furthermore, several studies show that ROS is mostly created in mitochondria and causes oxidative stress, which affects the biological process within cells (Kowalczyk et al. 2021).

Another study on hypocrellin B (HB), the well‐known photodynamic agent, was conducted to test its ability to be delivered more effectively and with more efficiency using nanoparticles made of natural and biodegradable polymer gelatin. With a near spherical shape and particle size, the HB loaded poly (ethylene glycol) modified gelatin nanoparticles (HB‐PEG‐GNP) exhibited distinctive optical features of PDT (Babu et al. 2012). Reactive oxygen species (ROS) were shown to be produced by photogeneration in HB‐PEG‐GNP photophysical investigations. Using Dalton's Lymphoma Ascites (DLA) cells, the nanoparticles were used for cellular absorption in vitro. After exposure to visible light, the nano‐formulation showed dose‐dependent phototoxicity. According to these investigations HB‐PEG‐GNP caused mitochondrial damage resulting into apoptotic cell death (Babu et al. 2012).

Additionally, a study was carried out to clarify the mechanism underlying HA's anticancer action as a possible PDT agent. The effectiveness of the potential drug was tested on A549 cells. Numerous studies have identified apoptosis—a gene‐mediated process of planned cell death—as a cellular signature route in PDT. Using biological markers of apoptotic cell death, the cell death mechanism that A549 cells experience upon HA and LED light was further investigated. Condensed chromatin, fragmented DNA, externalized phosphatidylserine all suggests that HA kills cancer cells by causing apoptotic cell death. Positive outcomes showed that PDT mediated by hypocrellin and its derivatives critically on mitochondria‐involved apoptosis (El‐Sikhry et al. 2010). Fu and Chu (1989) was the first to show that HA's photodynamic action may block hepatoma cells' mitochondrial ATPase in high‐voltage in vitro following high‐voltage sodium light. Furthermore, after PDT, hypocrellin B harmed the mitochondrial structure of ovarian cancer HO‐8910 cells, according to Jiang, Leung, et al. (2012). Zhao's group recently showed that PDT mediated by 17 (3‐amino‐1‐pentanesulfonic acid) substituted hypocrellin Schill base (PENSHB) lowered the inner membrane potential of the mitochondria, which released cytochrome *c* and caused apoptosis (Zhao et al. 2014).

Furthermore, HA was actively taken up into the cytoplasm and localized to mitochondria, as demonstrated by subcellular localization. El‐Sikhry and colleagues' investigation with the hypocrellin derivative SL01738 produced similar results regarding mitochondrial subcellular localization. The primary source of energy metabolism and the ATP production needed for cellular homeostasis is mitochondria (Kowaltowski et al. 2009; Vaghy 1979). For the first time, Otto Warburg noticed that compared to numerous non‐malignant cells, cancer cells make more lactate and consume more glucose. As a result, cancer cells' mitochondria are naturally more stressed by oxidative stress and more susceptible to apoptosis brought by ROS (Koppenol, Bounds, and Dang 2011). Future anticancer therapy may find it appealing to selectively target mitochondria of cancer cells (Cheng et al. 2012; Cheng et al. 2012). Finally, several studies have shown that light interaction excites HA in a triplet state, which can then combine directly with cell's substrate to generate free radicals, and oxidative stress. In summary, these studies indicate that hypocrellin qualifies as a potential photosensitizer to be used in PDT treatment of cancer cells. Additionally, this goes to show that the combination of hypocrellin derivatives with nano‐formulations play a vital role in drug delivery, stability, skin penetration and biocompatibility of the drug.

#### 2.5.1 ROS, p38/MAKP, PI3K/AKT Signaling Pathway, and Proapoptotic Proteins

The reactive oxygen species (ROS) system in cells is as a result of the contrasting processes of ROS production and scavenging. Since scavenging and production rates always occur in tandem in cells, shifting the balance in one direction would induce fast changes in ROS levels, which would trigger signals. Previous research demonstrates that in vivo ROS production by ROS producers (photo‐chemicals) combined with laser treatment, causes ROS to quickly localize in cellular components such as mitochondrial and cause fast damage of the cellular component and initiate apoptosis (Li et al. 2019; Cui et al. 2017).

Intracellular signaling networks like the mitogen‐activated protein (MAP) kinase pathways mediate cellular behavior in response to external stimuli (Rouse et al. 1994). MAP kinases function as focal points in response to a range of extracellular stimuli and are components of several signaling cascades. The body of research indicates that ROS build up can trigger MAPK signaling pathways (Gomez‐Lazaro et al. 2006; Xiong et al. 2015). In malignant melanoma, targeting MAKP signaling has proven to be a successful tactic (Marchetti et al. 2018).

The phosphatidylinositol 3‐kinase (PI3K)/protein kinase B (AKT) signaling pathway is one that regulates a variety of physiological functions within cells by activating downstream effector molecules that are essential for the cell cycle, growth, and proliferation (J. Rodon et al. 2013). Several research findings indicate that the PI3K/AKT signaling pathway may be linked to specific malignancies (Barra et al. 2018; Lu et al. 2018; Zhang, Zhou, and Gu 2018). Moreover, the thoughtful development of PI3K/AKT signaling pathway molecular targets represents a significant therapeutic avenue for these tumors.

The ROS, p38/MAPK, PI3K/AKP signaling systems and proapoptotic proteins work together in response to external stimuli in tumor cells. Studies have employed hypocrellin on cancer, which demonstrated that it triggers these signaling pathways (Figure 3). The well‐studied molecular pathways in human malignancies, MAPK, has been mentioned in several studies (Niu et al. 2020). The MAKP pathway has been assessed to learn more about the upstream mechanisms of hypocrellin A united with red light irradiation‐induced autophagy and apoptosis. The extracellular signal‐regulated kinase (ERK), p38, and c‐jun N‐terminal kinase (NK) are examples of the conventional MAPK pathway, and they are important regulators of cell migration, apoptotic cell death, and other processes (Sui et al. 2014). Numerous investigations have demonstrated the role of the JNK, ERK, and p38 pathways in controlling autophagy and apoptosis in response to stressors. It has also been shown that ROS mediates or induces the activation of the MAPK signal cascade (Hart et al. 2015).

The predominant mechanism of cell death is apoptosis. An early indicator of apoptosis is the caspase family, which is strongly connected to the cell death mechanism. There has been evidence that the control of apoptosis is influenced by the elevation of caspase‐3 activation. Niu T and associates found that upregulating the expression of activated caspase‐9 and caspase‐3 was caused by the combination of HA and red‐light irradiation. In the meanwhile, the Bcl‐2 family is also essential for controlling apoptosis, and a higher fraction of Bax/Bacl‐2 expression can trigger apoptosis. Therefore, the findings demonstrated that exposure of HA‐united red light increases the Bax/Bcl‐2 ratio (Niu et al. 2020). In summary, this section provides evidence that the signaling pathways co‐exists, and that hypocrellin can cause cell death.

## 3 Future Perspectives

From the typical simple tablet with uncontrolled release to system with enhanced bioavailability and less adverse effects, drug delivery technologies have come a long way. PDT‐based nanotechnology's benefits for cancer therapy using natural PS hypocrellin have been examined in this review. As the sections have explained, nonmedical technologies have proven to be beneficial in the detection and treatment of cancer, making it the most exemplary illness. It is highly likely that the field of nanomedicine and nano‐drug delivery systems will continue to be a focus of research and development for many years to come because they employ different kinds of nanoparticles to deliver precise dosages of medication to diseased cells, such as cancer or tumor, without interfering with the normal cell's physiology. The next line of inquiry would be to investigate materials with more reliable homogeneity and drug loading and release capabilities. Several studies cover significant amount of advancement in the use of metal‐based nanoparticles for diagnostic applications. Future studies in this field may lead to a larger use of nanomedicines. Examples of these metals are gold and silver, and their use in both diagnostic and therapy. The cost of nanomedicines is another issue that requires further investigation as they become more widespread. Ultimately, nanomedicine has its shortcomings, however the regulation of nanomedicines will improve in tandem with advancements in nanomedicine applications.

## 4 Conclusion

This review addresses current developments in nanomedicines, encompassing old and new drug delivery technologies and innovative diagnostic approaches. Initially, the main goals of using nano‐formulations in medicine were to improve the medications' solubility, absorption, bioavailability, and controlled release. Even though the search for pharmacologically active compounds in natural sources is less popular today than it was years ago, and even though the discovery of nano‐drugs is with uncertainty, improving the effectiveness of known natural bioactive compounds through nanotechnology has become a standard practice. Several studies using photosensitizers with nano‐formulations have been conducted, and hypocrellin has been used as one of the medications with potential for cancer therapy. Hypocrellin and several other nano‐formulations, especially nanoparticles, have demonstrated significant improvements in singlet oxygen production ability, stability, and water solubility. Moreover, hypocrellin and several nano‐formulations have a stronger capacity to react with biomolecules and trigger cell death pathways than free hypocrellin and that is because nano‐formulations have a large quantum yield of singlet oxygen (_1_O^2^). Now the potential for using hypocrellin in combination with nano‐formulations in clinical PDT therapy is substantial. The combination of nanotechnology and traditional techniques is a novel and promising way to treat melanoma; such combinations have shown good results. The nano‐formulations effectively and sparingly target intended melanoma cells because of their unique physical and biological characteristics. This as a result renews optimism for a more effective melanoma treatment plan with the least amount of harm. In summary, this review addressed the effective use of nano‐formulations used to improve drug delivery to targeted cells and reduce adverse effects. The use nano‐formulations have attracted so much interest to many researchers, and it has also been considered to treat other diseases other than cancer. The development of nanomedicines has made it possible for us to identify illnesses and even combine diagnosis and treatment.

## Author Contributions

**Precious Winterrose Gugu Nkosi:** investigation (lead), writing – original draft (lead), writing – review and editing (equal). **Rahul Chandran:** conceptualization (equal), funding acquisition (equal), project administration (equal), supervision (equal), writing – review and editing (equal). **Heidi Abrahamse:** funding acquisition (equal), resources (lead), supervision (supporting), writing – review and editing (equal).

## Conflicts of Interest

The authors declare no conflicts of interest.

## Related WIREs Articles

Recent advances in photodynamic therapy for cancer and infectious diseases

Nanocarriers in photodynamic therapy‐in vitro and in vivo studies

Innovative nanotheranostics: Smart nanoparticles based approach to overcome breast cancer stem cells mediated chemo‐ and radioresistances

Overcoming challenges in cancer treatment: Nano‐enabled photodynamic therapy as a viable solution

## Supporting information


**Figure S1.** Incidence of melanoma in selected European, American and African countries from the 1990s to 2020. Data are presented as age‐standardized rates in each country’s global standard population, in instances per 100,000 person‐years. (A) Diagram by Globocan 2018 shows the incidence and mortality rate across the world in both sexes, including all cancers for all ages. (B) Graph by Arnold et al. 2022 showing regions with the greatest melanoma incidence in 2020.
**Figure S2.** The diagram by Bertolotto 2013, shows the development and progression of melanoma. Melanoma develops from a pre‐existing nevus in 25% of instances, following a multistep process driven by a specific combination of genes. Before tumors and metastases to merge, cells must first acquire a series of genetic abnormalities.
**Figure S3.** The illustration by Scatena, Murtas, and Tomei (2021) depicts histological subtypes of melanoma: clinical‐pathological relationship of the various forms of melanoma. When an invasion takes place, a pigmented macule with uneven outlines (A) known as superficial spreading melanoma manifests as an atypical melanocyte proliferation in the papillary dermis (B). Nodular melanoma is an exophytic tumor that ranges in color from brown to black (C). Its major growth phase is vertical, and its melanocytes are spindle atypical or pigmented epithelioid, and they enter the reticular dermis (D). On sun‐damaged skin (E), lentiginous proliferation of atypical spindle melanocytes at the dermo‐epidermal junction with invasion into the papillary dermis is described as histologically as lentiginous proliferative melanoma; actinic damage and dermal elastosis are the surrounding skin (F). Atypical spindle melanocytes that are not pigmented may proliferate across the dermis (H) in an amelanotic nodule that is localized on the extremities (G).
**Figure S4.** The diagrams indicate the different ways by which hypocrellin can be incorporated to nano‐formulations using co polymers which are essential in the conjugation of hydrophilic and hydrophobic anticancer drugs. (A) Modified diagram HB‐PLGA NPs that were produced using the oil‐in‐water (O/W) emulsion‐solvent evaporation process by Lin et al. 2017. (B) Modified diagram by Feng et al. 2023 showing how PEG‐PLGA can be used in the encapsution/conjugation of hypocrellin B, this method uses precipitation to synthesize the bioconjugate.
**Table S1.** Summary of the efficiency of the various melanoma treatment modalities and their side effects by Hao et al. (2012).

## Data Availability

The authors have nothing to report.
