# Supplementary material for: Hypocrellin: A Natural Photosensitizer and Nano‐Formulation for Enhanced Molecular Targeting of PDT of Melanoma
Source: Wiley Interdiscip Rev Nanomed Nanobiotechnol. 2024 Nov 20;16(6):e1997. doi: 10.1002/wnan.1997 (PMC11579242; doi:10.1002/wnan.1997)
Supplement: Supplementary file 1 — Figure S1. Incidence of melanoma in selected European, American and African countries from the 1990s to 2020. Data are presented as age‐standardized rates in each country’s global standard population, in instances per 100,000 person‐years. (A) Diagram by Globocan 2018 shows the incidence and mortality rate across the world in both sexes, including all cancers for all ages. (B) Graph by Arnold et al. 2022 showing regions with the greatest melanoma incidence in 2020. Figure S2. The diagram by Bertolotto 2013, shows the development and progression of melanoma. Melanoma develops from a pre‐existing nevus in 25% of instances, following a multistep process driven by a specific combination of genes. Before tumors and metastases to merge, cells must first acquire a series of genetic abnormalities. Figure S3. The illustration by Scatena, Murtas, and Tomei (2021) depicts histological subtypes of melanoma: clinical‐pathological relationship of the various forms of melanoma. When an invasion takes place, a pigmented macule with uneven outlines (A) known as superficial spreading melanoma manifests as an atypical melanocyte proliferation in the papillary dermis (B). Nodular melanoma is an exophytic tumor that ranges in color from brown to black (C). Its major growth phase is vertical, and its melanocytes are spindle atypical or pigmented epithelioid, and they enter the reticular dermis (D). On sun‐damaged skin (E), lentiginous proliferation of atypical spindle melanocytes at the dermo‐epidermal junction with invasion into the papillary dermis is described as histologically as lentiginous proliferative melanoma; actinic damage and dermal elastosis are the surrounding skin (F). Atypical spindle melanocytes that are not pigmented may proliferate across the dermis (H) in an amelanotic nodule that is localized on the extremities (G). Figure S4. The diagrams indicate the different ways by which hypocrellin can be incorporated to nano‐formulations using co polymers which are essential i [file WNAN-16-e1997-s001.docx]

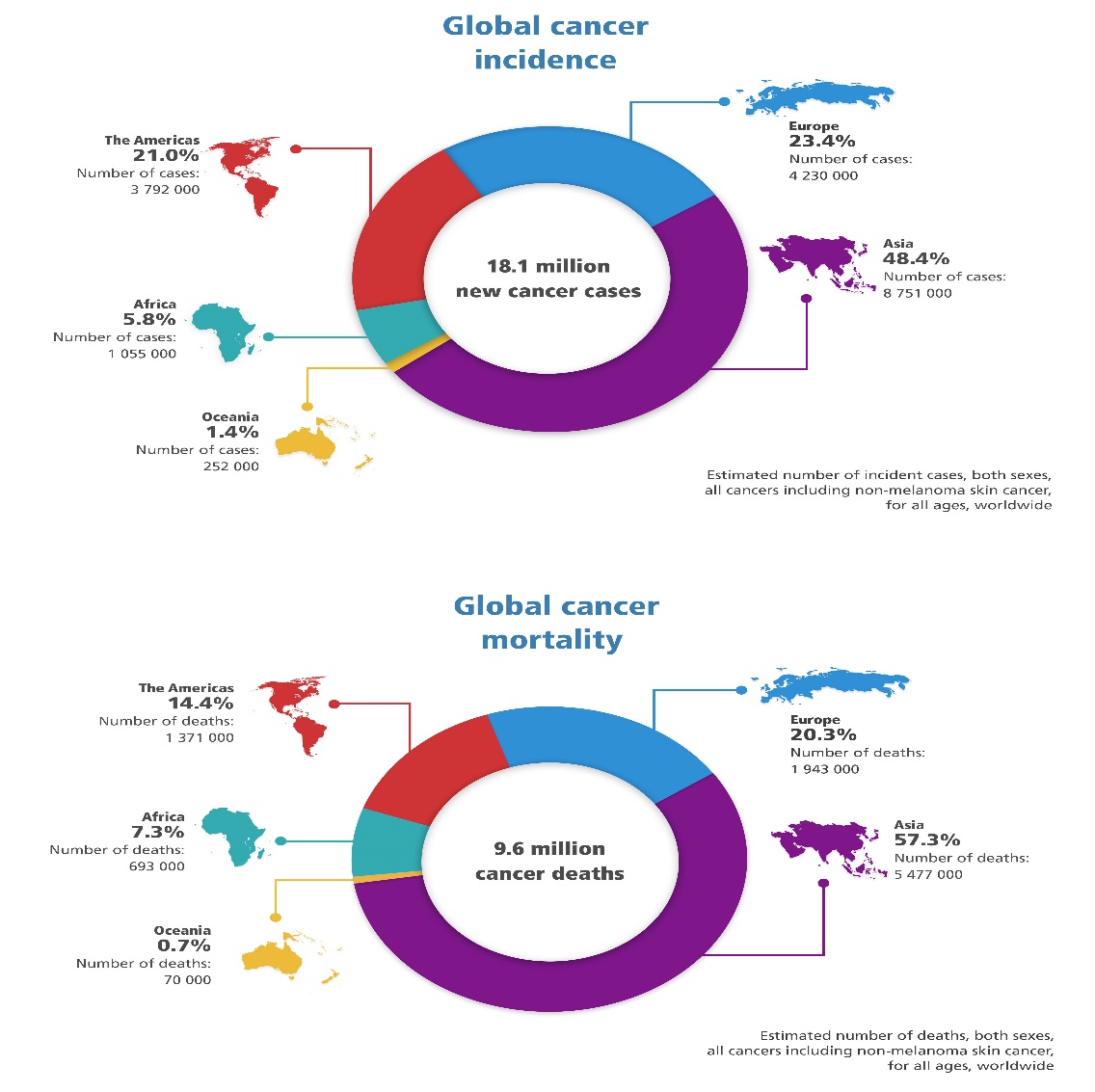


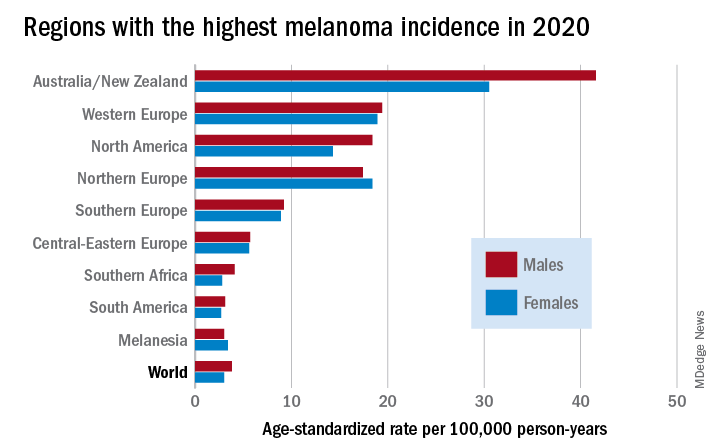


**Figure S1: Incidence of melanoma in selected European, American and African countries from the 1990s to 2020. Data is presented as age-standardized rates in each country’s global standard population, in instances per 100 000 person-years. (A) Diagram by Globocan 2018 shows the incidence and mortality rate across the world in both sexes, including all cancers for all ages. (B) Graph by Arnold et al., 2022 showing regions with the greatest melanoma incidence in 2020.**


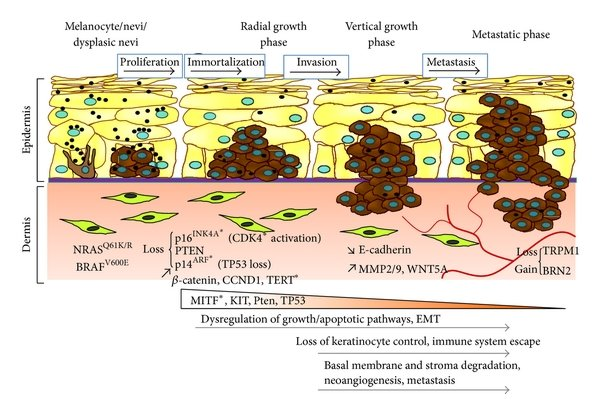


**Figure S2: The diagram by** **Bertolotto 2013, shows the development and progression of melanoma. Melanoma develops from a pre-existing nevus in 25% of instances, following a multistep process driven by a specific combination of genes. Before tumors and metastases to merge, cells must first acquire a series of genetic abnormalities.**


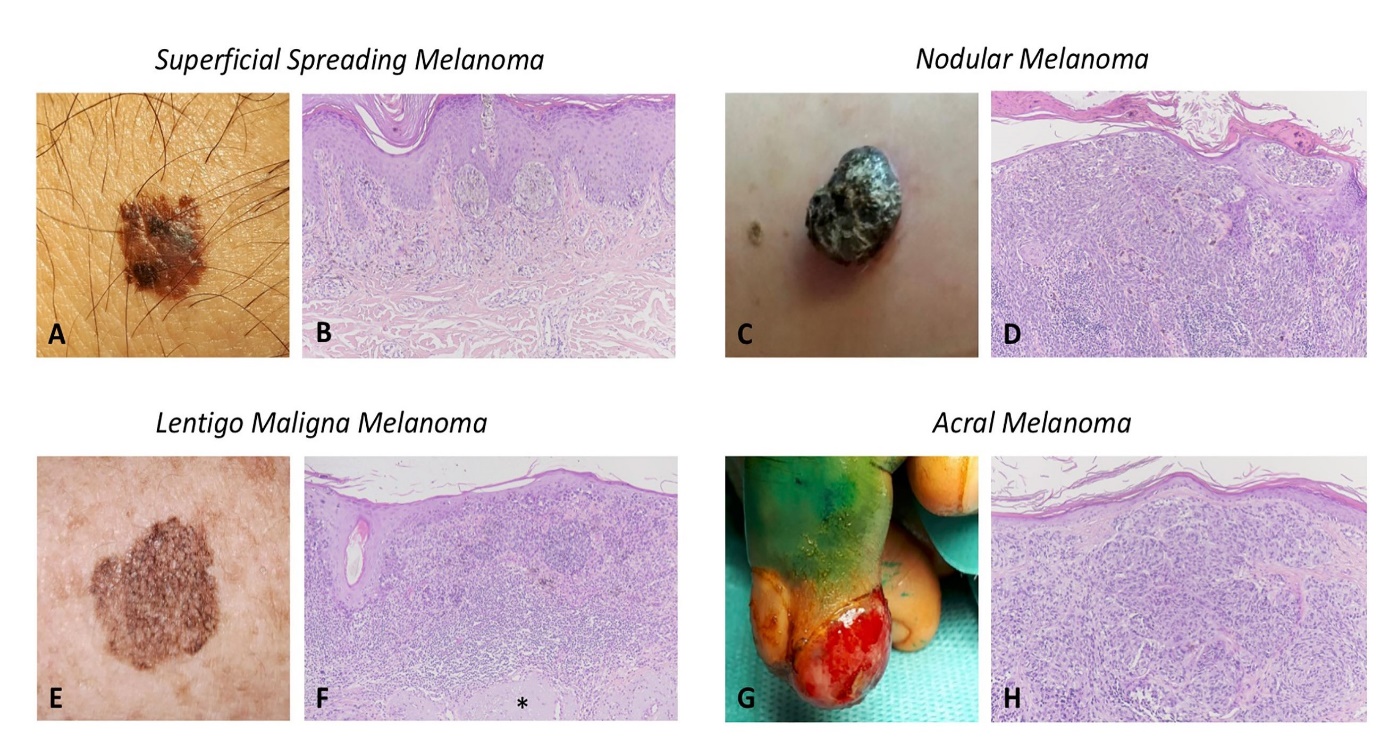


**Figure S3: The illustration by Scatena et al. (2021) depicts histological subtypes of melanoma: clinical-pathological relationship of the various forms of melanoma. When an invasion takes place, a pigmented macule with uneven outlines (A) known as superficial spreading melanoma manifests as an atypical melanocyte proliferation in the papillary dermis (B). Nodular melanoma is an exophytic tumor that ranges in color from brown to black (C). Its major growth phase is vertical, and its melanocytes are spindle atypical or pigmented epithelioid, and they enter the reticular dermis (D). On sun-damaged skin (E), lentiginous proliferation of atypical spindle melanocytes at the dermo-epidermal junction with invasion into the papillary dermis is described as histologically as lentiginous proliferative melanoma; actinic damage and dermal elastosis are the surrounding skin (F). Atypical spindle melanocytes that are not pigmented may proliferate across the dermis (H) in an amelanotic nodule that is localized on the extremities (G).**

**Table S1: Summary of the efficiency of the various melanoma treatment modalities and their side effects by Hao et al., 2021.**

| Treatment | Side Effects | Effectiveness | Limitations |
| --- | --- | --- | --- |
| Surgery | Pain | Is a successful cancer treatment it physically removes the tumor and any affected tissues. | Potential to miss stem cells, cells that are in pre-cancerous stages, or cells that have already metastasized, hence leading to reoccurrence. |
| Chemotherapy | Hair loss, intestinal damage and nausea. | Delivers medication through an IV to wipe out cancer cells | Cancer cells develop resistance to chemotherapy, not specific.  Healthy cells can be affected. |
| Targeted Therapy | Liver problems, diarrhoea, skin rash. | Prevents growth of cancer cells and blood vessels to the tumour | Cancer develops resistance. |
| Immunotherapy | Autoimmune effects | Activates your own immune system to help recognize and kill the cancer cells it can be effective. | Tailored and expensive |

**A**


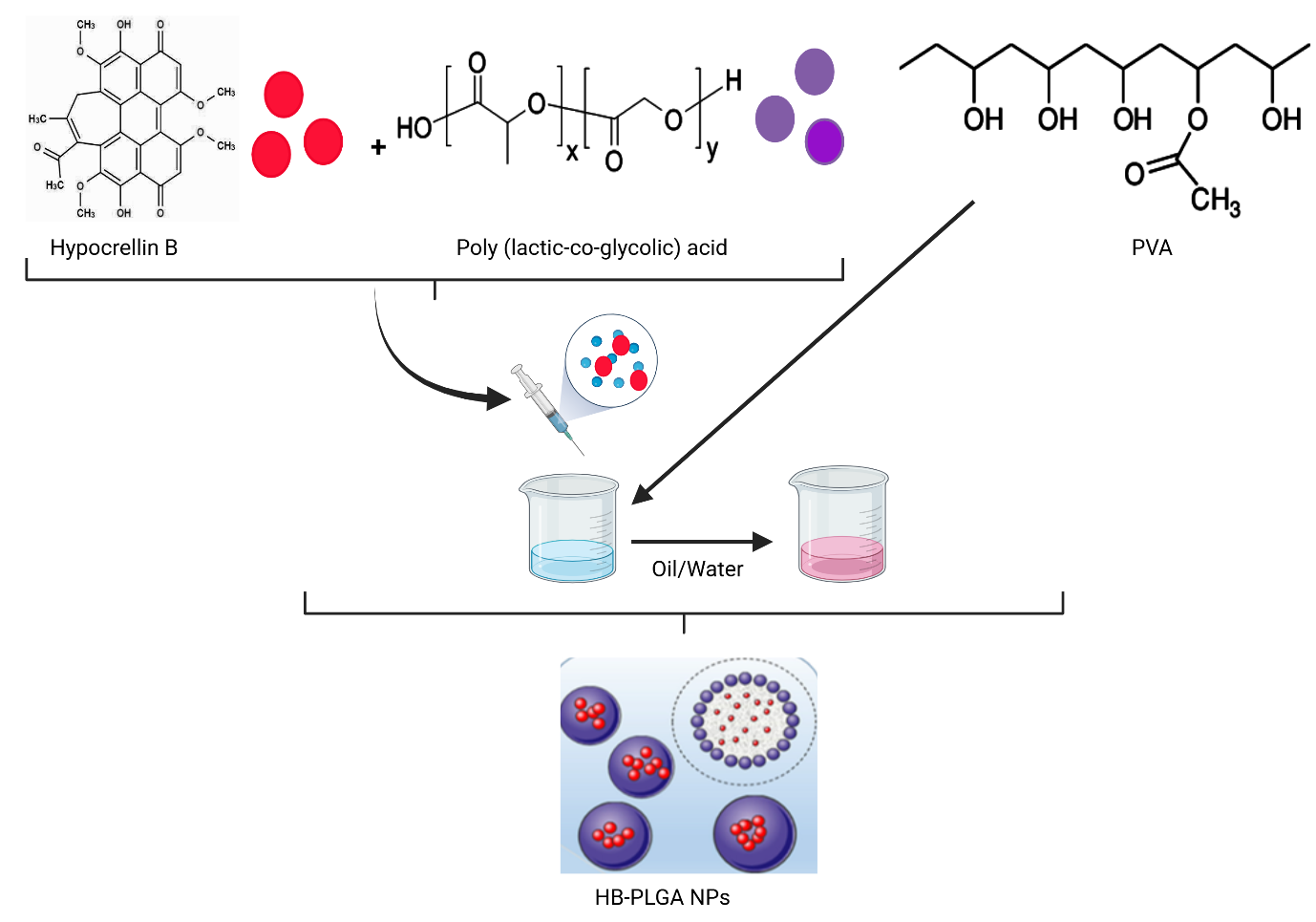


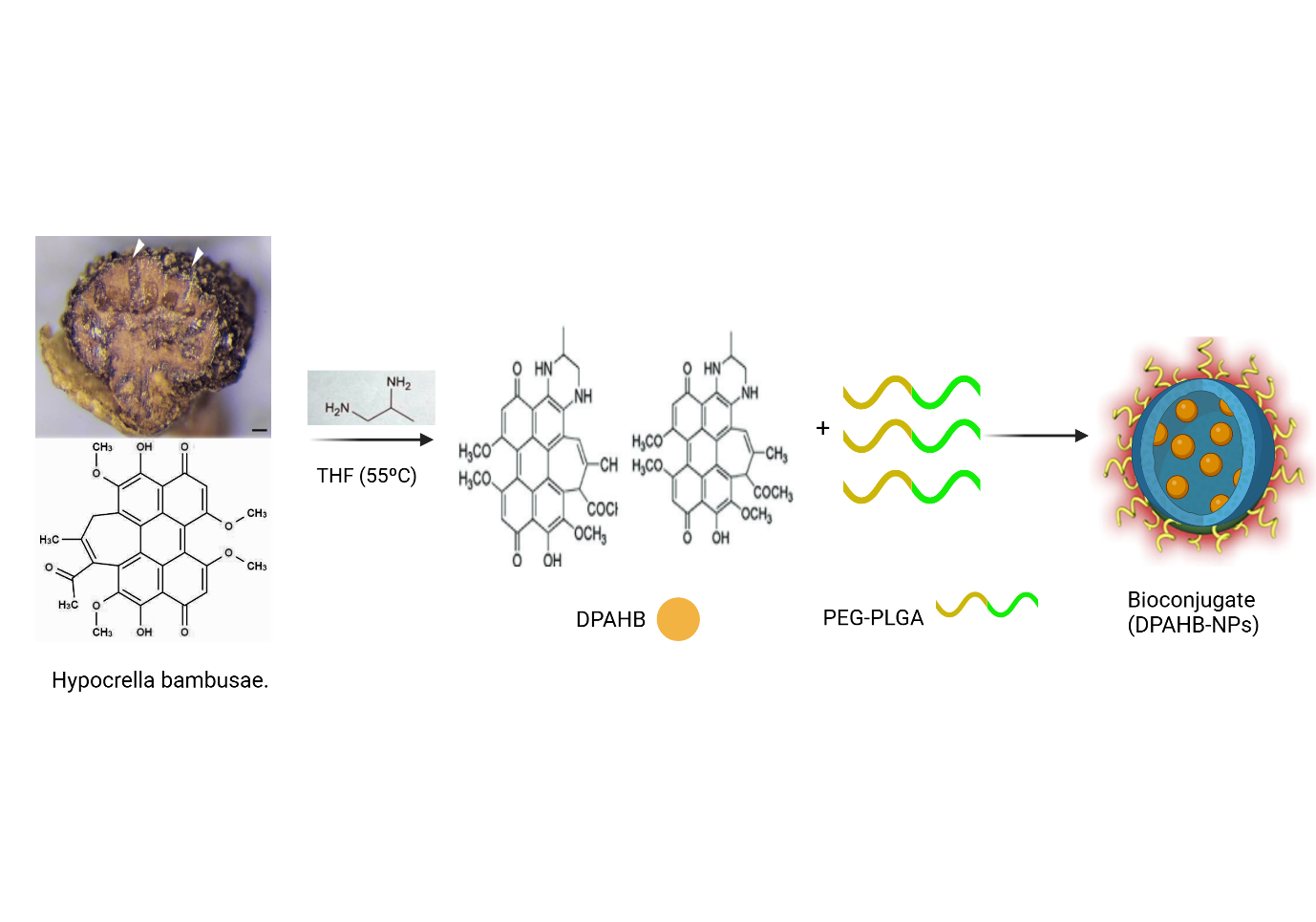


**B**

**Figure S4: The diagrams indicate the different ways by which hypocrellin can be incorporated to nano formulations using co polymers which are essential in the conjugation of hydrophilic and hydrophobic anticancer drugs. (A) Modified diagram HB-PLGA NPs that were produced using the oil-in-water (O/W) emulsion-solvent evaporation process by Lin et al., 2017. (B) Modified diagram by Feng et al., 2023 showing how PEG-PLGA can be used in the encapsution/conjugation of hypocrellin B, this method uses precipitation to synthesize the bioconjugate.**
